# Inhibition of Cancer Cell Migration and Glycolysis by Terahertz Wave Modulation via Altered Chromatin Accessibility

**DOI:** 10.34133/2022/9860679

**Published:** 2022-07-13

**Authors:** Lan Sun, Yangmei Li, Yun Yu, Peiliang Wang, Shengquan Zhu, Kaijie Wu, Yan Liu, Ruixing Wang, Li Min, Chao Chang

**Affiliations:** ^1^ Innovation Laboratory of Terahertz Biophysics, National Innovation Institute of Defense Technology, Beijing 100071 China; ^2^ School of Psychological and Cognitive Sciences, Peking University, Beijing 100871, China; ^3^ Aerospace Information Research Institute, School of Electronic, Electrical and Communication Engineering, University of the Chinese Academy of Sciences, Beijing 100049, China; ^4^ Key Laboratory of Electromagnetic Illumination and Sensing Technology, Chinese Academy of Sciences, Beijing 100190, China; ^5^ Department of Gastroenterology, Beijing Friendship Hospital, Capital Medical University, Beijing 100050, China; ^6^ Beijing Key Laboratory for Precancerous Lesion of Digestive Disease, National Clinical Research Center for Digestive Disease, Beijing 100171 China; ^7^ School of Physics, Peking University, Beijing 100871, China

## Abstract

Metastasis and metabolic disorders contribute to most cancer deaths and are potential drug targets in cancer treatment. However, corresponding drugs inevitably induce myeloid suppression and gastrointestinal toxicity. Here, we report a nonpharmaceutical and noninvasive electromagnetic intervention technique that exhibited long-term inhibition of cancer cells. Firstly, we revealed that optical radiation at the specific wavelength of 3.6  *μ*m (i.e., 83 THz) significantly increased binding affinity between DNA and histone via molecular dynamics simulations, providing a theoretical possibility for THz modulation- (THM-) based cancer cell intervention. Subsequent cell functional assays demonstrated that low-power 3.6  *μ*m THz wave could successfully inhibit cancer cell migration by 50% and reduce glycolysis by 60%. Then, mRNA sequencing and assays for transposase-accessible chromatin using sequencing (ATAC-seq) indicated that low-power THM at 3.6  *μ*m suppressed the genes associated with glycolysis and migration by reducing the chromatin accessibility of certain gene loci. Furthermore, THM at 3.6  *μ*m on HCT-116 cancer cells reduced the liver metastasis by 60% in a metastatic xenograft mouse model by splenic injection, successfully validated the inhibition of cancer cell migration by THM *in vivo*. Together, this work provides a new paradigm for electromagnetic irradiation-induced epigenetic changes and represents a theoretical basis for possible innovative therapeutic applications of THM as the future of cancer treatments.

## 1. Introduction

There is an estimation of 9,958,133 cancer-related deaths occurred in 2020, and most of these were caused by metastasis [ 1, 2]. Clinicians have developed various treatment approaches (e.g., postoperative chemotherapy and immunotherapy) to prevent and delay metastasis. However, chemotherapeutics can cause serious side effects, such as vomiting, diarrhea, and hair loss, due to unwanted cytotoxicity affecting normal “bystander” proliferating cells [ 3]. Immunotherapy provided very promising effects on inflamed tumors but failed to achieve favorable results on immune-excluded tumors and immune-desert tumors [ 4]. 

Many primary and metastatic tumors feature unusual energy metabolism as aerobic glycolysis is most favored by glucose homeostasis in cancer cells [ 5, 6]. The excess acidosis produced by glycolysis in the microenvironment induces adaptive changes in cancer cells, such as cell resistance to acid-induced toxicity [ 7, 8] and massive growth [ 9]. Additionally, many downstream genes of hypoxia-related signals participate in cancer cell motility and invasiveness [ 10]. For example, the activation of glucose transporter-3- (Glut3-) YAP signaling could reprogram cancer metabolism and thereby promote metastasis [ 11]. As a consequence, shifted energy metabolism in cancer cells and metastasis are intrinsically linked to each other and share common regulatory machinery [ 12]. Considering that significant and irreversible respiratory inhibition is a common feature of tumor metabolism [ 13], antimetabolites have been used in combination with other therapeutic regimens to improve the prognosis of cancer patients [ 14]. However, inevitable myeloid suppression and gastrointestinal toxicity also prevent the application and popularization of antimetabolites. Here, we aimed to develop a nonpharmaceutical approach to directly inhibit metastasis and metabolism of cancer cells without chemotoxic damage to the whole body. 

Biomacromolecules, once irritated, absorb part or all of the energy of incoming electromagnetic (EM) waves depending on the incident frequency [ 15]. Since the generalized terahertz (THz) band (0.5-100 THz) partially overlaps with the vibrational spectrum of biomolecules [ 16], the terahertz wave could largely enhance bond vibrations of biomolecules such as twisting, stretching, and bending via resonant excitation [ 17, 18]. Nonetheless, early research on biological effects of optical stimulation focuses on the near-infrared region, which facilitates strong absorption of incoming EM energy by water and transduction of it into thermal heat [ 19– 21]. While the heat changes the transmembrane capacitive charge or ion channel activities and hence evokes neural responses, it inevitably induces a transient increase in local temperature [ 22]. On the other hand, terahertz wave modulation (THM) is treated as a promising nondamaging electromagnetic radiation intervention approach [ 23, 24]. Investigation on the nonthermal biological effects of THz irradiation has attracted extensive attention of both opticists and biologists [ 25]. Furthermore, the THz waves with low photon energy hardly cause ionizing effects and thus would not damage the genome integrity as other radiation intervention approaches might [ 26– 29]. 

Many studies verified that THz waves with different radiation frequencies, intensities, and time of exposure shed different effects on the functions (e.g., gene expression, synaptic transmission, and immune cytokines release) of the nervous system [ 30– 32]. Also, the THz waves could cause structural changes of macromolecules (e.g., proteins, lipid membranes) in living cells [ 24, 33]. However, while most of these studies disclosed the relations between altered biological functions and different THz radiations, they are in lack of in-depth insights into the molecular and physical mechanisms. Our team has long been dedicated to THM and its nonthermal effects. We revealed greatly enhanced permeability of ion channels [ 34] and accelerated unwinding of DNA duplexes [ 35] and reduced receptor-ligand binding [ 36] under specific THM stimuli. Later, we experimentally achieved reversibly shortened action potential (AP) waveforms [ 37] and modulated the activity of neurons in targeted brain cortical areas by THM [ 38]. All these results indicate the regulatory role of THM on the biomolecules. However, the effects of terahertz waves on cancer cells are largely unexplored. Previous studies have mainly focused on the detection of tumors by terahertz, and the mechanism of terahertz effect on tumor cells is not systematically demonstrated from the combination of molecular structure theory and biological experiments. 

**Scheme 1:**
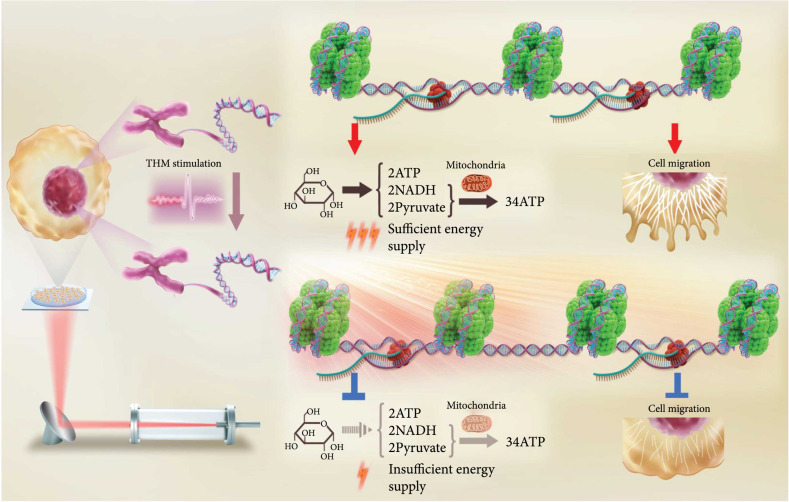
Schematic of the suppression effects of THM on cancer cells. THM at a specific wavelength (3.6  *μ*m) significantly inhibited the migration and glycolysis of cancer cells by altering the chromatin accessibility of genes.

In this work, we explored whether the terahertz field could regulate interactions between histones and DNA via molecular dynamics (MD) simulations and achieved significantly increased binding affinity under a THM at 3.6  *μ*m. Based on this finding, we designed and built a frequency-tunable THM light source and conducted a systematic evaluation of THM-induced biological effects on two cancer cell lines to verify the long-term and fundamental biological effects. The results showed that 3.6  *μ*m THM could significantly inhibit the migration and glycolysis of cancer cells. In a metastatic xenograft mouse model, we further reconfirmed that THM significantly exhibited the metastasis of colorectal cancer. In combination with multiomics studies, we identified that 3.6  *μ*m THM should suppress the migration and glycolysis in cancer cells by altering genomic chromatin accessibility as we predicted by MD simulations (Scheme 1). 

## 2. Results

### 2.1. THM at 3.6  *μ*m Largely Enhances Binding between Histones and DNA in MD Simulations 

**Figure 1:**
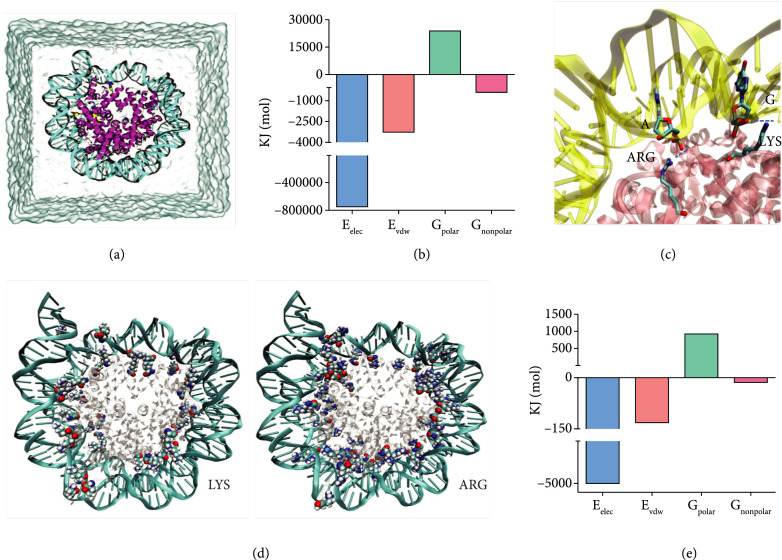
The binding free energy between histones and DNA before and after being irritated by THz wave. (a) Schematic of the simulated nucleosome core particle with histones in the middle wrapped by DNA helices and immersed in NaCl solution. (b) Four decomposed energetic terms of the total binding free energy between protein and DNA in the baseline case. (c) Two typical salt bridges (blue dashed lines) formed between PO4- of adenine (A)/guanine (G) bases and the positively charged side chains of arginine and lysine residues. (d) Lysine and arginine moieties of the histones located within 7 Å of the DNA. (e) Variations of the four energetic terms under 3.64  *μ*m THM.

We firstly explored the possibility of THz wave-regulated gene expression by means of molecular dynamics simulations. To this end, we investigated how THM affected histone-DNA affinity and screened the most likely effective wavelength. The simulation system was constructed with a nucleosome core immersed in 150 mM NaCl solution (Figure 1(a)). In the baseline case without THM, the free energy of binding between histone octamers and DNA was -728,544 kJ/mol, which denoted a remarkably strong binding. After decomposition into four energetic terms (Figure 1(b)), $E_{\text{elec}}$ originating from electrostatic interactions overwhelmingly dominated and reached up to -748,744 kJ/mol, while the other three terms were relatively trivial. Since lysine and arginine amino groups of histones are positively charged and phosphate groups in DNA are negatively charged, electrostatic attractions (via H-bonds or more specifically salt bridges) between them dominate binding interactions (Figures 1(c) and 1(d)). The van der Waals interactions and nonpolar solvation also contribute positively to binding affinity, whereas polar solvation with positive free energy implies a negative effect. 

Since electrostatic attractions between the DNA and histones play a dominant role in DNA-histone binding, and the H-bond connections mainly exist in the form of N—H···O, an illumination source in resonance with N-H bond vibrations will probably alter related nonbonded interactions (Suppl. Figure S1). This has been discussed in detail in the Supporting Information. Therein, we have demonstrated three central vibrational frequencies of N-H bonds, among which the one at 2,746 cm ^-1^ corresponding to the strongly H-bonded N-H vibrations turns out to be most effective to alter the histone-DNA interactions (Suppl. Figure S2). When the nucleosome was irritated by a 2746 cm ^-1^ (3.6  *μ*m) THz wave, the binding free energy decreased significantly by 4,206 kJ/mol, which suggested an enhanced affinity within the complex. The enhancement mainly derived from alteration to electrostatic contributions, as the electrostatic free energy decreased by 4,999 kJ/mol in response to the irradiation (Figure 1(e)). Apparently, since the THM at 2,746 cm ^-1^ (3.6  *μ*m) could remarkably enhance binding between histones and DNA, it possesses great potential of regulating gene expression and cell phenotype. 

### 2.2. THM at 3.6  *μ*m Inhibits Cancer Cell Migration and Glycolysis at a Low Power of 0.3 mW 

Infrared and THz spectroscopy has been proposed as a noninvasive tool in cancer diagnosis and imaging owing to its excellent tissue permeability [ 39]. Additionally, some studies have already demonstrated the potential of near-infrared illumination applied in cancer therapy, which mainly focused on the development of nanocarriers that can encapsulate drugs for rapid photorelease [ 40, 41]. As we have already proved that THM could be an efficient approach to directly regulate binding between histones and DNA in MD simulation (Figure 1), it would be important to further explore the detailed functional influence on cancer cells. 

**Figure 2:**
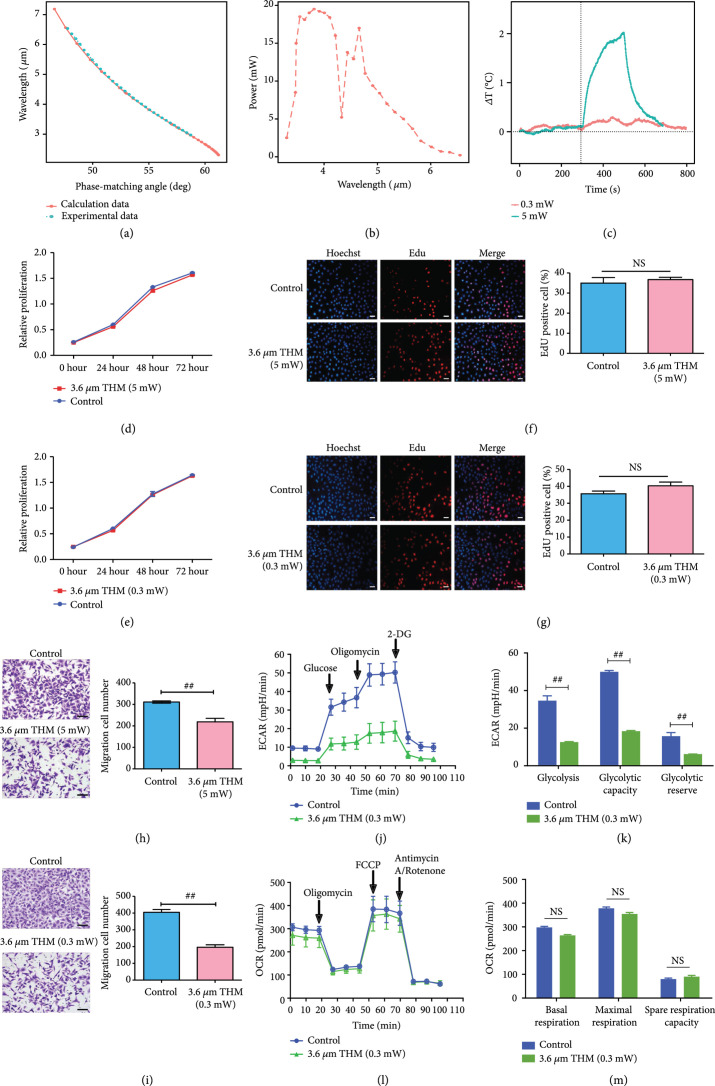
THM (3.6  *μ*m) profoundly inhibited cancer cell migration and glycolysis at a power of 0.3 mW. (a) The red line represents the relationship between the theoretically calculated phase matching angle and the output wavelength, while the green line represents the measured values. (b) Different phase matching angles correspond to the average power of infrared light generated at different wavelengths. (c) Temperature changes after THM for 300 s ( P=5/0.3 mW; λ=3.6 *μ*m). (d and e) MTS results showing the effect of different THM power ( P=5/0.3 mW) on SW480 cell proliferation. (f and g) EdU results showing the effects of different THM power ( P=5/0.3 mW) on SW480 cell growth. Scale bar, 100  *μ*m (left). (h and i) Transwell migration assay results showing the effects of different THM power ( P=5/0.3 mW) on SW480 cell migration. Scale bar, 100  *μ*m (left). (j) Effects of THM ( P=0.3 mW) on extracellular acidification rate. (k) THM downregulated maximum glycolysis, glycolytic metabolic capacity, and glycolytic reserve ability of SW480 cells. (l) Effects of THM ( P=0.3 mW) on oxygen consumption rate. (m) THM did not influence basal respiratory capacity, maximum respiratory capacity, or respiratory reserve capacity of SW480 cells. All data are shown as means±SEM , ^#^ P<0.05 , and ^##^ P<0.01 . NS: not significant.

Thus, here we designed and built a frequency-tunable light source to evaluate the long-term and fundamental biological effects of THM on cancer cells. The phase-matching condition is satisfied by controlling the angle between the crystal and the 1,064 nm pump light. Different phase-matching angles correspond to different wavelengths, ranging from 2.3 to 7.2  *μ*m (Figure 2(a)). The maximum amount of power also varies with wavelength. When the wavelength is set to 3.6  *μ*m according to the parameters recommended by the MD simulations (Figure 1), the maximum power is about 15 mW (Figure 2(b)). There is nearly 67% attenuation of the THz wave penetrating through the petri dish, and we could inflict a maximum power of 5 mW on the cancer cells culturing in the Petri dish. Correspondingly, the minimum power we could enforce on the cell is 0.3 mW. A temperature-specific probe showed that the temperature change resulting from THM (3.6  *μ*m and 0.3 mW) was almost undetectable and much lower than that of 5 mW illumination at the same wavelength (Figure 2(c)). 

SW480 cells were irradiated with THM at 5 mW and 0.3 mW, respectively. This illumination was repeated at 10 Hz with a pulse width of 10 ns to ensure effective energy delivery. Cell proliferation assays suggested that 3.6  *μ*m THM did not affect cell proliferation ( P>0.05 , Figures 2(d) and 2(e)). Similar results were obtained in EdU staining (Figures 2(f) and 2(g)). It follows that 3.6  *μ*m THM did not perturb cell growth. 

As for cell migration evaluations, we found that 5 mW THM at 3.6  *μ*m could successfully inhibit cancer cell migration ( P<0.01 , Figure 2(h)). Likewise, THM at 3.6  *μ*m successfully maintained the 50% cell migration inhibition rate at 0.3 mW ( P<0.01 , Figure 2(i)). Assays involving another cancer cell line, HCT-8, exhibited the same results (Suppl. Figure 3a-c). Thus, we concluded that THM (3.6  *μ*m and 0.3 mW) strongly inhibited cancer cell migration. 

Compared to normal cells, cancer cells have remarkably elevated metabolic requirements [ 42]. To meet the demand for more energy, cancer cells have to consume additional glucose and divert nutrients into macromolecular synthesis pathways to produce lactic acid and support their proliferation and metastasis [ 43]. With a drastic change in the expression of metabolism-associated genes, which play critical roles in cell growth and survival, glycogen metabolism in cancer cells is altered [ 44]. Here, we further explored whether THM ( P=0.3 mW, λ=3.6 *μ*m) inhibited glycolysis or oxidative phosphorylation in cancer cells. Firstly, we performed extracellular acidification rate (ECAR) assays to test glycolysis levels under THM. These revealed that cells exposed to THM ( P=0.3 mW, λ=3.6 *μ*m) were significantly inhibited in terms of extracellular acidification rate (Figure 2(j)). These data showed that THM could effectively reduce the level of maximum glycolysis, glycolytic capacity, and glycolytic reserve ability by 60% (Figure 2(k)). We further tested whether THz wave ( λ=3.6 *μ*m) could affect oxidative phosphorylation levels in cancer cells. Compared to a control group, the basal respiration, maximum respiratory capacity, and respiratory reserve capacity of the cancer cells failed to exhibit significant change under THM (Figures 2(l) and 2(m)). In summary, THM ( P=0.3 mW, λ=3.6 *μ*m) only decreased glycolysis, but not oxidative phosphorylation in cancer cells. Even though the energy supply is crucial for cell growth and proliferation, it is not necessarily a rate-limiting factor. Here, we found that THM significantly inhibited ECAR but did not alter the proliferation of cancer cells. The reduced ATP by the hampered ECAR process could not decrease the proliferation of cancer cells since the sustaining proliferative signaling was not much changed by THM-induced changes of energy supply. Previous studies revealed that THz wave (3.5-5.7  *μ*m) was hardly absorbed by water and could exert nonthermal, long-distance, and reversible modulatory effects on neuronal excitability [ 45]. The low THM power adopted in this study also largely excluded nonspecific thermal effects as compared to other studies concerning the biological effects of THM [ 46]. To the best of our knowledge, this is the first study to demonstrate the nonthermal tumor-suppressor effects of THM with such a low-power laser light source. Additionally, this result indicated the feasibility of applying THM in cancer treatment, considering that 0.3 mW of power could be successfully delivered into the site of a tumor even under a high attenuation rate. 

### 2.3. THM at 3.6  *μ*m Altered the Expression Levels of Genes Governing Cell Migration and Metabolism 

**Figure 3:**
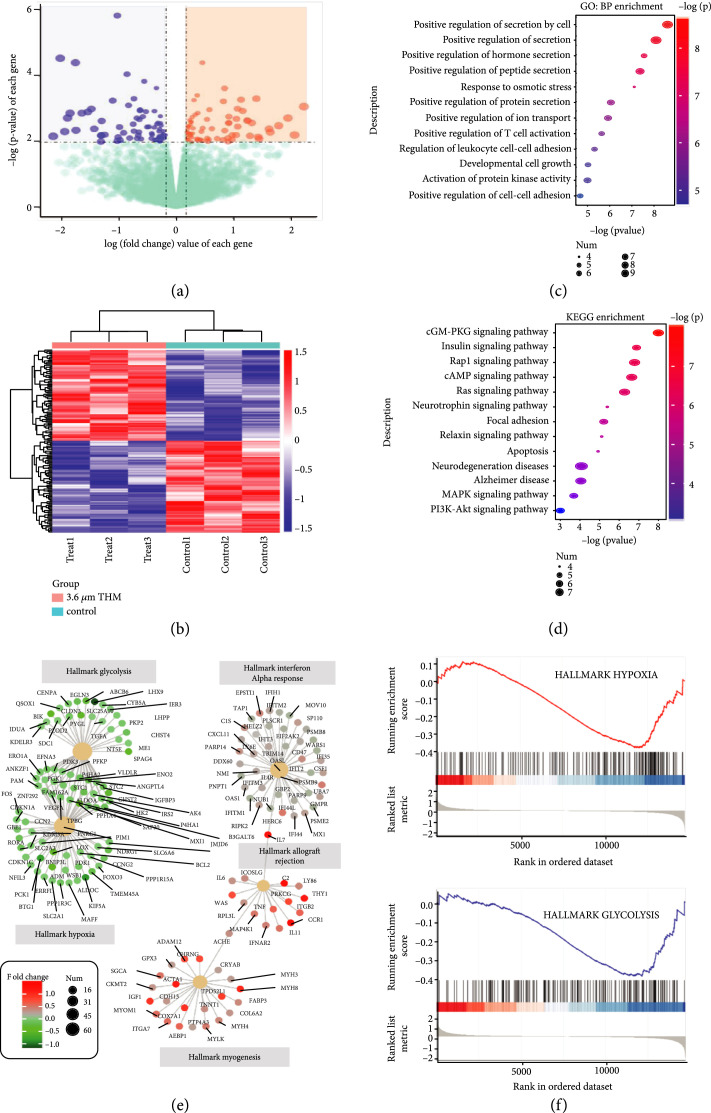
RNA-seq revealed differentially expressed genes under 3.6  *μ*m THM. (a) Identification of differentially expressed genes by RNA-seq induced by THM (genes with a log10-fold change>0.176 and a P value < 0.01 are colored red, while those with a log10-fold change<−0.176 and a P value less than 0.01 are colored blue; others are colored green). (b) Heat map of significantly differentially expressed genes between the control and 3.6  *μ*m THM groups (with P<0.01 ), with red indicating upregulation and blue indicating downregulation. Hierarchical clustering of transcriptional profiles in the control group and 3.6  *μ*m THM group. The color key represents FPKM normalized log2 transformed counts, and each row represents a gene. (c) GO analysis of differentially expressed genes. Each row represents a BP term; the size indicates the number of genes enriched; the color intensity indicates the -log ( P value). (d) KEGG pathway analysis of differentially expressed genes (DEGs). Each row represents a KEGG term; the size indicates the number of genes enriched; the color indicates the -log ( P value). (e) Top five GSEA pathways. The color intensity indicates the fold change, and the size indicates the number of genes enriched. (f) GSEA analysis revealed that glycolysis- and hypoxia-associated genes were inhibited.

To reveal the panorama of changes involving gene expression in cancer cells under low-power 3.6  *μ*m THM, SW480 cells after illumination were subjected to RNA sequencing along with untreated controls. A total of 145 genes were identified as being differentially expressed ( $\left|\text{fold}\text{change}\right|\geq 1.5$ , P<0.01 ), among which 72 genes were upregulated and 73 were downregulated by THM (Figure 3(a)). These differentially expressed genes (DEGs) largely discriminated between the THM group and a control group (Figure 3(b)), indicating that low-power 3.6  *μ*m THM resulted in a drastic change in gene expression. 

In the Gene Ontology (GO) biological process enrichment analysis, the altered genes were enriched for the positive regulation of secretion, positive regulation of cell-cell adhesion, and activation of protein kinase (Figure 3(c)). Furthermore, Kyoto Encyclopedia of Genes and Genomes (KEGG) enrichment analysis also identified a variety of signaling pathways, such as MAPK signaling, PI3K-Akt signaling, focal adhesion, and Ras signaling (Figure 3(d)). 

In gene set enrichment analysis (GSEA), the top five enriched hallmark pathways included interferon- *α* response, glycolysis, and hypoxia (Figure 3(e)). Further analysis suggested that both glucose metabolism and hypoxia signaling were reduced in the THM group (Figure 3(f)). Thus, we concluded that low-power 3.6  *μ*m THM stimulation could lead to dramatic changes in gene expression, by which the processes of glucose metabolism, hypoxia signaling, and ultimately the migratory ability of cancer cells were inhibited. 

Evidence suggests that many downstream genes of hypoxia and hypoxia-related signals participate in cancer cell motility and invasiveness [ 10]. Moreover, regulatory mechanisms underlying energy metabolism and metastasis in cancer are intrinsically linked to each other and share common regulatory machinery [ 12]. As we found in GO/KEGG enrichment analysis and GSEA, HIF- *α* and glucose metabolism-related genes were drastically changed by THM. Thus, the THM inhibitory effects on cancer cell invasion could be due to the suppression of the shared regulatory factors (e.g., HIF-1 *α*) and related downstream glucose pathway regulation of the Warburg effect [ 47]. 

Dynamic control of cell-cell interactions by THM has also attracted attention for the development of cell-based therapies. Chang et al. reported that THM reduces the cellular migratory and invasive abilities of breast cancer cells by remodeling focal adhesion molecules [ 46]. Here, our data supported that the expression of cell-cell adhesion regulatory genes was shifted by THM, providing an in-depth explanation of the molecular basis for THM inhibition of invasion and metastasis of cancer cells. 

### 2.4. THM at 3.6  *μ*m Altered Chromatin Accessibility 

**Figure 4:**
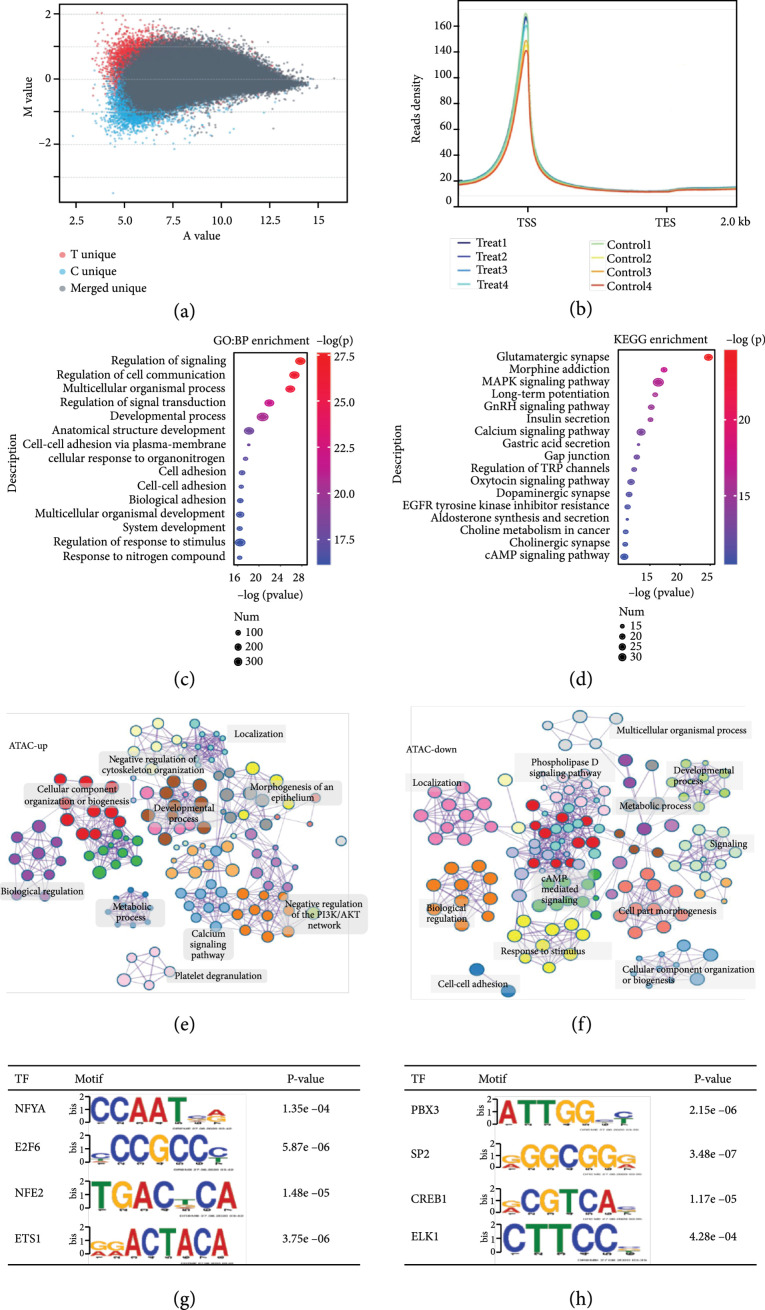
Genomic chromatin accessibility altered by THM (3.6  *μ*m and 0.3 mW). (a) Identification of differentially accessible genes by ATAC-seq induced by THM. The horizontal axis indicates the normalized and log2-transformed average degree of the opening of each peak, the vertical axis indicates the difference on the normalized and log2-transformed average degree of the opening of each peak resulted by THM (specific peaks only detected in the THM group are colored red; specific peaks only detected in the control group are colored blue; common peaks detected in both groups are colored grey). (b) Density distribution of reads in chromosomes (the horizontal axis represents the normalized gene location, and the vertical axis represents the read density). TSS: transcription start site; TES: transcription stop site. (c) GO analysis of differential peaks. Each row represents a BP term; the size indicates the number of genes enriched; the color indicates the -log ( P value). (d) KEGG pathway analysis of differential peaks. Each row represents a KEGG term; the size indicates the number of genes enriched; the color intensity indicates the -log ( P value). (e) Cytoscape pathway analysis of differentially opened peaks. (f) Cytoscape pathway analysis of differentially closed peaks. (g and h) TFs binding to the regions with differentially opened and closed peaks.

The spatiotemporal heterogeneity of gene expression is determined by the different accessibility of TFs to the binding, unwinding, and transcriptional extension of the target DNA. Genes in a dormant state are highly agglutinated with histones, and only gene elements located in chromatin in an extended form can be bound by TFs. It has been widely recognized that chromatin structure defines a scenario where interactions between TFs and their regulatory regions occur [ 48]. Our MD simulations revealed that THM (3.6  *μ*m and 0.3 mW) altered the binding between histones and DNA without changing its primary structure or the DNA unwinding process. To verify the hypothesis that THM enhances binding involving histones and DNA, we performed ATAC-seq, a method for genome-wide mapping of chromatin accessibility [ 49]. In ATAC-seq analysis, 85,068 peaks were identified as being differentially open/closed in the THM group, among which 54,959 peaks indicated open chromatin, and 30,109 peaks represented closed regions under THM as compared to controls (Figure 4(a)). The peak density was located at the transcription start site (TSS) (Figure 4(b)). For GO enrichment analysis, the top-ranked differentially open/closed peaks were enriched in the regulation of responses to stimuli, developmental processes, biological adhesion, and cell adhesion, among others (Figure 4(c)). For KEGG enrichment analysis, MAPK, calcium, and cAMP signaling pathways were enriched (Figure 4(d)). The results indicated that THM induced a large number of unique ATAC peaks as open or closed. The differentially open/closed peaks changed after THM were located on genes associated with the regulation of responses to stimuli, cell metabolism, and cell adhesion. The altered migration activity induced by THM could be due to changes in chromatin accessibility of certain genes. 

**Figure 5:**
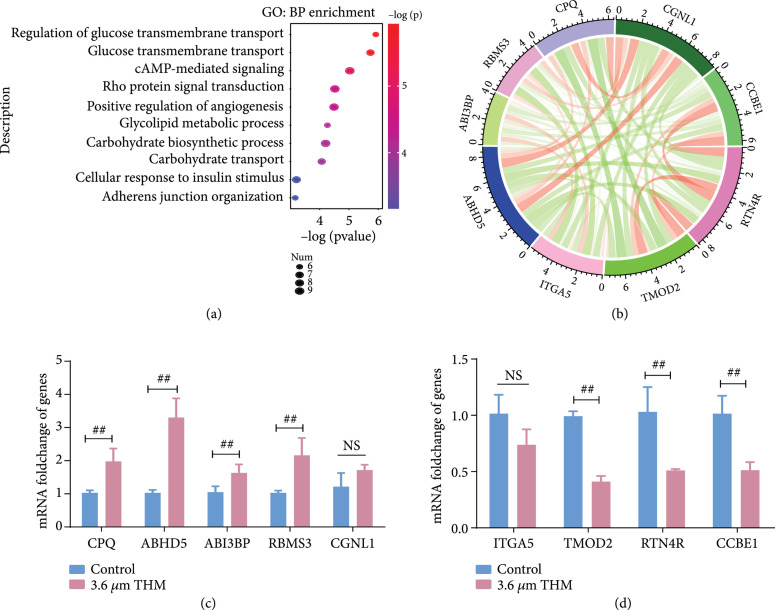
Altered genomic chromatin accessibility resulted in expression changes of genes governing the migration and glycolysis of cancer cells. (a) GO analysis of differential peaks (the genes exhibited >1.5-fold change in mRNA levels with changes in chromatin accessibility peaks). Each row represents a BP term; the size indicates the number of genes enriched; the color indicates the -log ( P value). (b) Circos plot displaying interconnectivity among genes altered by THM ( λ=3.6 *μ*m; P=0.3 mW). (c) RT-qPCR confirmed the increased levels of CPQ, ABHD5, AB13BP, RBMS3, and CGNL1 under THM. (d) RT-qPCR confirmed the decreased levels of ITGA5, TMOD2, RTN4R, and CCBE1 under THM. Results are expressed as means±SEM ( ^##^ P<0.01 ). NS: not significant.

For genes altered from close to open peaks (ATAC-up), we visualized interactions among enriched pathways using Cytoscape. The pathways were mostly associated with localization, calcium signaling, and metabolic process (Figure 4(e)). For those genes altered from open to closed peaks (ATAC-down), altered pathways were enriched in responses to stimuli, cell part morphogenesis, localization, and metabolic process (Figure 4(f)). Thus, both open and closed peaks were closely linked to pathways associated with metabolic processes and cellular localization. We also explored transcription factor (TF) binding to these regions with changed peaks by motif enrichment and identified NFYA, E2F6, PBX3, ETS1, and ELK1 as the main regulators affected by THM (Figures 4(g) and 4(h)). As reported in previous studies, ETS1 promoted endothelial cell migration and invasion by activating the transcription of integrins and MMPs [ 50– 52]. Correspondingly, in our ATAC-seq and RNA-seq results, both the chromatin accessibility and transcription of those genes were inhibited (Figure 5(d)). Similar changes were identified in the target genes of ELK1. Therefore, here we suggested that altered chromatin accessibility of target genes of ETS1 and ELK1 could at least partially explain the inhibition of migration and glucose metabolism after THM. 

To assess whether the observed changes in chromatin accessibility resulted in changes in gene expression, we further investigated the expression of genes with changes in chromatin accessibility peaks. Among the 1,418 genes with changes in chromatin accessibility peaks, 499 exhibited a >1.5-fold change in mRNA levels. We further explored those genes by GO enrichment. These 499 genes were mainly associated with glucose transport, glycolipid metabolism, and adherens junction organization, etc., which were largely in accordance with the results of the above cellular functional assays (Figure 5(a)). Among these genes, we further verified the expression levels of CCBE1, ABHD5, and ITGA5, etc. by RT-qPCR. The results suggested that the expression of these genes was significantly altered by THM (Figures 5(b)– 5(d)). 

### 2.5. THM at 3.6  *μ*m Decreased the Liver Metastasis of Cancer Cells *In Vivo*

As the theoretical basis for possible therapeutic applications of THM in cancer treatments had been illustrated, we further explored the potential effects of THM on cancer cell migration *in vivo*. We constructed a mouse model of liver metastasis by the splenic injection of HCT-116 colorectal cancer cells and recorded the metastasis foci after 4 weeks. 

**Figure 6:**
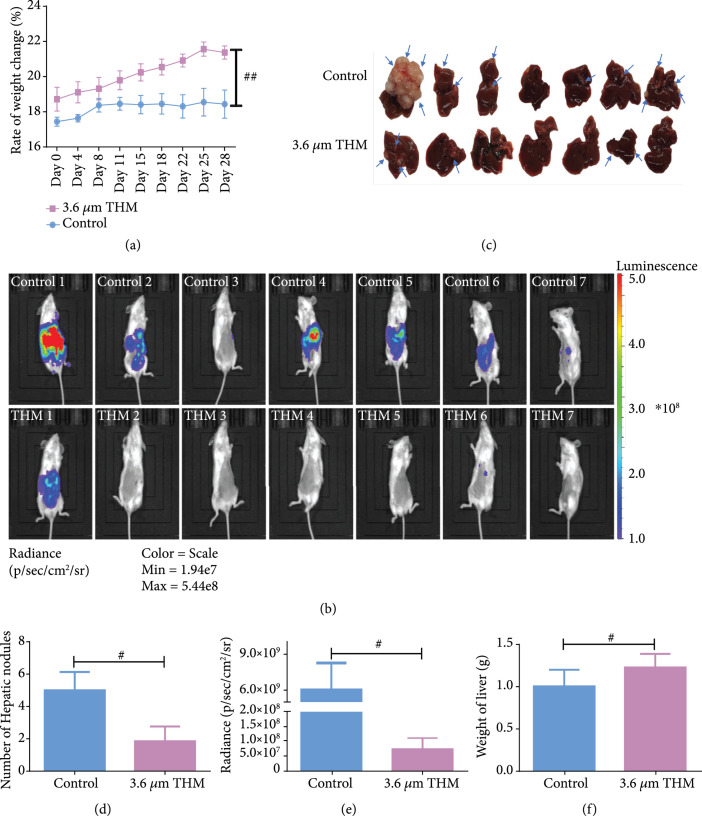
THM at 3.6  *μ*m decreased the liver metastasis of cancer cells in vivo. (a) Body weight of the mice after splenic injection of HCT-116 cancer cells. (b) The bioluminescence images of all mice 4 weeks after splenic injection. The upper panel showed the mice of control group, while the lower panel displayed the THM group. The bioluminescence signals are presented in color: blue for the lowest and red for the highest intensity. (c) The images of the livers with and without metastasis harvested 4 weeks after splenic injection. The blue arrows indicated the metastasis foci. (d) The comparison on the number of hepatic nodules between the THM group and the control group. (e) The comparison on the bioluminescence signals between the two groups. (f) The comparison on the weight of liver between the two groups ( n=7 for each group, ^#^ P<0.05 ).

Generally, we found that cancer cells pretreated with THM ( λ=3.6 *μ*m; P=0.3 mW) before splenic injection were much less possible to form a successful metastasis. The increase of body weight was higher in the mice of the THM group compared to those of the control group, suggesting that those mice were less affected by cancer cachexia (Figure 6(a)). Both the quantification of bioluminescence signals and anatomical appearance revealed that cancer cells pretreated with THM formed much fewer metastasis foci as compared to the control cancer cells (Figures 6(b) and 6(c)). Precisely, the THM reduced the average number of hepatic nodules from 5 to 2 (Figure 6(d)) and decrease the bioluminescence signal from $6\times 10^{9}$ to $5\times 10^{7}$ (Figure 6(e)). Moreover, the average weight of the liver was 1.0 g in the control group and 1.2 g in the THM group (Figure 6(f)), which was consistent with the results on body weight (Figure 6(a)). Thus, we concluded that 3.6  *μ*m THz on HCT-116 cancer cells reduced the liver metastasis by 60% in a metastatic xenograft mouse model by splenic injection, successfully validated the inhibition of cancer cell migration by THM *in vivo*. 

## 3. Discussion

In summary, we elucidated that low-power THM could successfully inhibit the migration and glycolysis of cancer cells by altering genomic chromatin accessibility, which suggests a potential application of THM in cancer treatment. Additionally, our findings, where THM altered chromatin accessibility, provided a new paradigm for electromagnetic waves resulting in epigenetic changes. However, the detailed mechanism on the THM-mediated change of 3D genome structure should still be investigated. Also, this work will expand our understanding of the long-term biological regulatory effects of electromagnetic illumination, representing a theoretical basis for various innovative therapeutic applications of THM. Nevertheless, a pilot clinical study of THM treatment on cancer patients would be urgently needed to evaluate its clinical application ability in the near future.

## 4. Materials and Methods

### 4.1. Molecular Dynamics Simulations

To evaluate the effects of THz stimulus on binding between DNA and histones, we constructed a nucleosome complex and conducted a series of MD simulations using GROMACS 2019.4 [ 53]. The initial neutral system consisted of 129,400 atoms, including the nucleosome core particle, 35,813 water molecules, 219 sodium ions, and four chloride ions. The simulation box size is 12.3nm×8.2nm×12.4nm in x , y , and z directions. The crystal structure of nucleosome core particles [ 54] is derived from the Research Collaboratory for Structural Bioinformatics (RCSB) Protein Data Bank (PDB entry: 2CV5) and is an octamer tightly wrapped by two superhelical turns of 146 bp of DNA. The octamer is composed of two copies of each of the four histone proteins, H2A, H2B, H3, and H4. In the simulations, amber14sb_parmbsc1 force fields (FFs) were used, which can provide high-quality descriptions of protein-DNA complexes [ 55, 56]. The simulation time step was set as low as 1.0 femtoseconds, which took into account of the high frequency field stimulation and were also compromised between the computational accuracy and efficiency. The simulation system was initially temperature coupled with the V-rescale thermostat to a bath of 310.15 K (same as the experiment temperature) and then pressure coupled with the Berendsen barostat to 1 bar for 5 ns where the protein and DNA were position restrained with decreasing force constants. Afterwards, the system without any restraints was further relaxed for 50 ns to get equilibrated, where the pressure coupling method was changed to the more accurate Parrinello-Rahman method. Following this, the atom trajectories were recorded for 40 ns for further calculations. For cases with THz irradiation, an electric field $E\left(t\right)=A\cdot u\cdot \cos\left(2\pi ft+\varphi \right)$ is applied as an alternative to the EM field, as the effect of the magnetic component on the atoms is negligible for atomic velocities significantly lower than the speed of light, c . Here, A represents the maximum amplitude of the electric field, which is 2.4 V/nm for all irritated cases; u and φ represent the polarization direction and the initial phase, which are set to (0, 0, 1) and 0, respectively. The illumination wave number k is related to the wave frequency f by k=2πf/c . 

The binding free energy $∆G_{\text{binding}}$ between histones and DNA in solution reads as

(1)∆Gbinding=Gnucleosome−Ghistones+GDNA,

where $G_{\text{nucleosome}}$ is the total free energy of the nucleosome core particle and $G_{\text{histones}}$ and $G_{\text{DNA}}$ are the free energies of individual histones and DNA in the solvent, respectively. The free energy can be decomposed into

(2)G=Ebonded+Evdw+Eelec+Gpolar+Gnonpolar−TS,

where the first three energetic terms represent the bonded (bond, angle, and dihedral), van der Waals, and electrostatic interactions, respectively. The fourth and fifth terms refer to the polar/electrostatic and nonpolar contributions to the solvation free energy, respectively. The last parameter denotes entropic contribution. For a single trajectory, $∆E_{\text{bonded}}$ is defined as zero. Because the net entropic contribution is usually small and debatable in improving free energy estimates, it is neglected during the calculations. Therefore, the binding free energy only considers four energetic terms, that is, the van der Waals free energy $∆E_{\text{vdw}}$ , electrostatic free energy $∆E_{\text{elec}}$ , polar/electrostatic solvation free energy $∆G_{\text{polar}}$ , and nonelectrostatic solvation free energy $∆G_{\text{nonpolar}}$ . For simplicity, we leave the symbol ∆ in the following: the g_mmpbsa tool can also offer residue contributions to the total binding energy, which facilitates the identification of important residues involved in molecular associations. During trajectory processing, snapshots extracted every 1.0 ns were considered uncorrelated and were used to calculate the averages of the binding energies based on the bootstrap method. 

### 4.2. Design and Fabrication of a Tunable THM Emitter for Cellular Functional Assays

The pumping laser was a diode-pumped Nd:YAG electrooptic Q-switched laser (Q-smart 850, Quantel, Lannion, FR). In these experiments, the repetition frequency of the laser was 10 Hz. The maximum pulse energy reached 850 mJ. The pulse width was approximately 10 ns, and the divergence angle of the laser was <0.5 mrad. The optical parametric oscillator (OPO) was generated by the gain crystal, barium selenium gallium [ 57]. 

The scanning lens was an off-axis parabolic mirror (OPM) fixed on an x‐y scanning platform. The scanning platform was produced by Thorlabs (Type: NRT 100/M, Newton, New Jersey, US), and the controller was BSC203. Based on the Kinesi operation program offered by Thorlabs, we compiled an x‐y two-dimensional scanning program. The effect of light path scanning and sample immobility was achieved by controlling the beam to hit the position of the off-axis paraboloid mirror. 

In these experiments, the light spot diameter on the focal plane of the OPM was 1 mm after culture dish, so we set the speed of axis x to 0.5 mm/s to ensure that most cells could obtain two seconds of infrared illumination. The step of axis y was 0.5 mm so that all cells could be radiated. On the focal plane, we fixed a supporting bracket to place a small cell incubator to offer a suitable environment for the culture dish. The bottom of the small cell incubator was made of a special material that blocks infrared light. There was a 2.5 cm diameter circular area at the bottom. Therefore, only infrared light illuminating the round part could enter the cell culture. 

### 4.3. Evaluation of Possible Temperature Changes by THM

The possible temperature changes induced by THM using a specific probe were evaluated (Physitemp, Needle Microprobes, MT-29/5, Clifton, New Jersey, US). Powerlab was used to record the temperature every 0.01 seconds, and the mean of every 10 data points was calculated to reduce random perturbations. A probe was fixed on the z -axis robotic arm of the stereotactic machine and precisely operated under the monitoring of a stereo microscope to contact the cells at the bottom of the culture dish. Before THM, the temperature of the cells was recorded for 300 s to evaluate possible environmental perturbations. THM lasted for 180 s, during which the temperature was fully recorded. Then, the THM was removed and recovery of the temperature was monitored for >300 s. To ensure the accurate evaluation of the THz power delivered to the cancer cells, we measured the power at the same position in each trial. 

### 4.4. Cell Lines, Culture Conditions, and THM Stimulation

SW480 and HCT-8 are two common human colon cancer cell lines and were purchased from the American Type Culture Collection (ATCC) (Manassas, VA, USA). SW480 and HCT-8 cells were cultured in a humidified atmosphere of 5% CO _2_ at 37°C. The medium was 90% DEME (Corning, Glendale, AZ, USA) or DMEM (Corning) containing 10% fetal bovine serum (FBS) (Gibco, Grand Island, NY. USA), 100 units/ml of penicillin-streptomycin (Invitrogen, Carlsbad, CA). 

To scan the entire area of the Petri dish, a 2D moving platform was used to control the scanning direction and step size, and the scanning speed and direction were set for scanning one by one. The scanning process of each Petri dish was performed for 30 min. We regarded the center of the cell culture dish as the midpoint. During the entire process, including transfer and placement of the cells receiving THM, a control group with the same Petri dishes was kept in the same environment. To ensure that the difference between the control group and the experimental group is only the presence or absence of terahertz illumination, the dishes of both groups were kept in a temporary device with the same atmosphere and temperature as the incubator when the dishes of the experimental group received scanning.

### 4.5. Assessment of Cell Viability and Proliferation Rate

Cells were seeded at a density of 3,000 cells/well in 96-well plates. After 24 h of incubation, cells were treated with MTS reagent for 0, 24, 48, and 72 h. After incubation for 2 h at 37°C, an enzyme-labeled meter (SpectraMax M3, Molecular Devices, San Jose, CA, USA) was used to assess cell viability.

The cells were attached to 96-well plates and then treated with EdU (Cell-Light™ EdU Apollo567 In Vitro Imaging Kit, Ribobio, BJ, CN) solution at 37C°C for 2 h, followed by fixation in 4% formaldehyde for 30 min. The cells were then treated with Hoechst 33342 for 30 min for nuclear staining. Finally, cell proliferation was observed using a fluorescence microscope (Olympus, DP72, Shinjuku-ku, TKY, JPN).

### 4.6. Cell Migration Assays

Migration assays were performed using a Transwell chamber (8  *μ*m, Corning) with Matrigel (Corning). The cells were seeded into the upper chamber in culture medium containing 1% FBS. The lower wells were filled with complete medium and served as a chemoattractant. Next, the upper surface was removed, and cells were allowed to migrate through the pores. After 24 h, migrated cells were fixed and stained. The cells in three randomly selected fields were counted under a fluorescence microscope and images were captured. 

### 4.7. Metabolic Analysis

The oxygen consumption rate (OCR) and extracellular acidification rate (ECAR) were measured using a Seahorse XFe24 Flux Analyzer (Seahorse Bioscience, North Billerica, MA, USA) according to the manufacturer’s instructions. Briefly, the cells were treated with PET-16 for 24 h and subjected to metabolic testing. For glycolysis stress assay test (Kit103020, Seahorse Bioscience, North Billerica, MA, USA), carbonyl cyanide 4-(trifluoromethoxy) phenylhydrazone (FCCP) and oligomycin were diluted in XFp media and loaded into an accompanying cartridge to achieve final concentrations of 10 mM. For mitochondrial stress assays (Mito Stress Test Kit103015, Seahorse Bioscience, North Billerica, MA, USA), 1  *μ*M FCCP and 0.5  *μ*M rotenone/antimycin A were diluted in XFp media and loaded into an accompanying cartridge to achieve final concentrations. Upon completion of the Seahorse XFp Flux analysis, the cells were lysed to calculate protein concentrations. The results were normalized to protein abundance in the corresponding wells. Data are representative of three biological replicates. 

### 4.8. RNA Isolation and Library mRNA Sequencing

Total RNA from cancer cells was extracted using TRIzol reagent (Thermo Fisher, Waltham, MA, USA) according to the manufacturer’s instructions. RNA samples were then digested with RNase-free DNase I (Invitrogen, Carlsbad, CA, USA) to eliminate residual genomic DNA. The quality of the cDNA library was checked using an Agilent 2100 Bioanalyzer (Agilent Technologies, Santa Clara, CA, USA). The cDNA library was sequenced on a paired-end flow cell using an Illumina Hiseq 4000 platform (Illumina, San Diego, CA, USA). Raw reads were preprocessed using FastQC software [ 58]. Transcript expression levels were estimated using fragments per kilobase per million reads (FPKM) values and quantified using RSEM software [ 59]. 

Differential expression analysis of two groups (three biological replicates per condition) was performed using the limma package. The resulting P values were obtained using Benjamini and Hochberg’s approach for controlling the false discovery rate. Genes with a P value ≤ 0.01 and $\left|-\text{fold}\text{change}\right|\geq 1.5$ were assigned as differentially expressed genes (DEGs). GO analysis was classified into three subgroups: biological process (BP), cellular component (CC), and molecular function (MF). For BP enrichment analysis, GO terms with corrected P values < 0.05 were considered significantly enriched. Kyoto Encyclopedia of Genes and Genomes (KEGG) enrichment of DEGs was implemented by the hypergeometric test, in which the P value was adjusted by multiple comparisons as q values. KEGG terms with q<0.05 were considered to be significantly enriched [ 60]. For GSEA, a false discovery rate $\left(\text{FDR}\right)<0.25$ was considered statistically significant [ 61]. 

### 4.9. ATAC-seq

ATAC-seq was performed as described previously [ 62]. Sorted cells (50,000 cells per cell type centrifuged at 500×g for 5 min, 4°C) were prepared for ATAC-seq transposition. Cell pellets were rinsed with 50  *μ*L cold 1 × PBS buffer and centrifuged at 500×g for 5 min, 4°C. Gently pipet up and down to resuspend the cell pellet in 50  *μ*L of cold lysis buffer. Centrifuge immediately at 500×g for 5 min, 4°C. After nuclei preparation, the pellets were kept on ice and gently resuspended in transposition reaction mix. Following purification, isolated DNA samples were transferred to 96-well PCR plates and amplified using PCR. The quality of the purified libraries was also assessed using a Bioanalyzer High Sensitivity DNA Analysis kit (Agilent). 

Differentially accessible regions of chromatin were identified and classified by applying Bowtie2 [ 63] and MACS2 [ 64] to count data output from the ATAC-seq pipeline. Peaks were selected for inclusion in the count matrix if they were identified as statistically significant (based on the MACS Poisson model described above) in any input sample, and any overlapping peaks were merged. Differential peaks are identified using nominal P values < 0.05. 

Once differential peaks are identified, we mapped these peaks to the genes they located in. Further GO and KEGG enrichment analysis were also performed and terms with P<0.05 were considered significantly enriched [ 65]. Cytoscape software (v.3.8.2) was used to visualize the functional interaction networks constructed by the genes altered in ATAC-seq [ 66]. TF binding motif analysis of ATAC-seq was performed using MEME [ 67] and Dreme [ 68]. 

### 4.10. Real-Time Quantitative RT-PCR

Total RNA was extracted by TRNzol Universal Reagent (Tiangen, BJ, China). After assessing RNA quality using a NanoDrop One spectrophotometer (Thermo Fisher), the RNA samples were reverse-transcribed with PrimeScript RT Master Mix (TaKaRa Bio, Japan), and quantitative real-time PCR was performed using PowerUp™ SYBR™ Green Master Mix in a real-time PCR system (Applied Biosystems, Carlsbad, CA). The primer sequences are listed in Suppl. Table 1. Quantification of each mRNA was carried out with glyceraldehyde 3-phosphate dehydrogenase (GAPDH) as reference genes. 

### 4.11. Animal Experiments and Bioluminescence Imaging

Four- to five-week-old female NOD SCID immunodeficient mice (CYAGEN, SH, China) were housed within a specific pathogen free (SPF) environment at a professional animal laboratory (temperature 22-26°C, 10/14-hour light/dark cycles with humidity 40-70%). All mice were allowed ad libitum access to standard food and water. All animal experimental procedures were in strict accordance with the National Institutes of Health Guide for the Care and Use of Laboratory Animals (NIH, Publications No. 80-23) and were conducted with approval from the Institutional Animal Care and Use Committee (IACUC) of Beijing friendship hospital.

After adaptive feeding for seven days, all animals were randomly divided into two groups: the THM group and the control group. Briefly, colorectal cancer cell line HCT-116 expressing the luciferase enzyme gene Luc (HCT116-Luc) was used (HEYUAN, SH, China) for intrasplenic injection to construct a metastatic xenograft mouse model. Mice were inoculated with HCT116-Luc cells ( $3\times 10^{6}$ cells per mouse) stimulated by 3.6  *μ*m THM for 30 min (THM group, n=7 ) or an isovolumetric intrasplenic injection of untreated HCT116-Luc cells (control group, n=7 ). The intrasplenic injections of HCT116-Luc tumor cells were performed under aseptic conditions as previously reported [ 69]. All surgical procedures were performed in strict accordance with institutional guidelines. The weight of each animal was monitored twice a week. 

After 28 days, all mice were subjected to bioluminescence imaging. Each mouse received an intraperitoneal weight-adapted injection of D-luciferin (150  *μ*L) at a dose of 150  *μ*g/g before imaging. The mice were placed in the bioluminescence signal detection chamber for imaging 20 min after injection. Bioluminescence imaging was conducted using IVIS Lumina Series III Imaging System (PerkinElmer, Waltham, MA). Regions of interest (ROI) of the same size and shape were applied to all mice images to assess total flux (photons per sec) in ROI. At the experimental endpoint, animals were euthanized, and liver tissue was weighted and subjected to anatomical examination. 

### 4.12. Statistical Analysis

Data were analyzed using SPSS v.19.0 (SPSS, IBM Corp, Armonk, NY), GraphPad Prism 5.0, and R software. Data represent means±SEM of at least three independent experiments. Comparisons between two groups were performed using two-sided Student’s t -test. Differences were considered statistically significant at a P value < 0.05. 

## Data Availability

The data that support the findings of this study are available from the corresponding author upon reasonable request.

## Supplementary Materials

Supplementary Materials Supplementary Methods. Figure S1: hydrogen bonds within the DNA and salt bridges between histones and the DNA. Figure S2: mechanism underlying the enhanced histones-DNA binding. Figure S3: effects of THM (0.3 mW) on HCT-8 cell. Supplementary Table 1: RT-qPCR primers used in this study.
