# Supplementary material for: Inhibition of Cancer Cell Migration and Glycolysis by Terahertz Wave Modulation via Altered Chromatin Accessibility
Source: Research (Wash D C). 2022 Jul 13;2022:9860679. doi: 10.34133/2022/9860679 (PMC11697589; doi:10.34133/2022/9860679)
Supplement: Supplementary Materials — Supplementary Methods. Figure S1: hydrogen bonds within the DNA and salt bridges between histones and the DNA. Figure S2: mechanism underlying the enhanced histones-DNA binding. Figure S3: effects of THM (0.3 mW) on HCT-8 cell. Supplementary Table 1: RT-qPCR primers used in this study. [file 9860679.f1.docx]

**S****upplementary Information**

**Inhibition of Cancer Cell** **Migration and Glycolysis by** **Terahertz Wave Modulation via Altered Chromatin Accessibility**

Short Title: THM Inhibition on Cancer Migration and Glycolysis

Lan Sun^1,2,§^, Yangmei Li^1,§^, Yun Yu^1^, Peiliang Wang^1,3,4^, Shengquan Zhu^5,6^,

Kaijie Wu^1^, Yan Liu^1^, Ruixing Wang^1^, Li Min^5,6,*^, Chao Chang^7,1,*^

^1^Innovation Laboratory of Terahertz Biophysics, National Innovation Institute of Defense Technology, Beijing, 100071; ^2^School of Psychological and Cognitive Sciences, Peking University, Beijing, 100871; ^3^Aerospace Information Research Institute; School of Electronic, Electrical and Communication Engineering, University of the Chinese Academy of Sciences, Beijing 100049; ^4^Key Laboratory of Electromagnetic Illumination and Sensing Technology, Chinese Academy of sciences, Beijing 100190; ^5^Department of Gastroenterology, Beijing Friendship Hospital, Capital Medical University, Beijing, 100050; ^6^Beijing Key Laboratory for Precancerous Lesion of Digestive Disease; National Clinical Research Center for Digestive Disease, Beijing, 100171; ^7^School of Physics, Peking University, Beijing, 100871, China.

* *joint corresponding senior authors*; ^§^*joint first authors*.

Correspondence should be addressed to:

Prof. Chao Chang (gwyzlzssb@pku.edu.cn) and Prof. Li Min (minli@ccmu.edu.cn).

**Supplementary Methods**

1. **Unit conversion**

In the manuscript, we have chosen to use wavelength $\lambda$, frequency *f*, and wavenumber *k* to describe the Terahertz wave according to its usage scenarios. These three quantities have a simple relation as follows:

$\lambda=c/f=2\pi/k.$ (1)

where *c* represents the speed of light. When incorporating the common units, equation (1) can be expressed as:

$\lambda(\mu m)=300/f(\mathrm{THz})={10}^{4}/k(\mathrm{cm}^{-1}).$ (2)

1. **Frequency estimation of effective THM on nucleosome affinity**

Note that the strong H-bonds connecting the histones and DNA mainly exist in the form of N—H···O (Suppl. Figure 1a). It follows that a radiation source in resonance with N-H bond vibrations will probably alters the relating non-bonded interactions. The N-H related H-bonds are also prevalent in the individual DNA and histones. For instance, the strong H-bonds hold the paired pyrimidine-purine nucleotide bases together in the form of N—H···O and N—H···N (Suppl. Figure 1b-c). As to histones, the N—H···O bonds connect the amino acides as in the Suppl. Figure 1d.

In order to estimate the radiation frequency adopted in subsequent experiments, we calculated the absorption spectrum of a DNA fragment and compared it to the given experimental spectrum [1, 2].

The absorption spectrum can be calculated by Fourier transforming the electrical flux-flux autocorrelation function [3, 4]:

$I(\omega)=\int_{0}^{\infty} dt<\boldsymbol{C}(0).\boldsymbol{C}(t)>e^{-i\omega t}$ (3)

where $\boldsymbol{C}(t)=\sum_{j=1}^{N} q_{j}\boldsymbol{v}_{j}(t)$ represents summation of the electrical fluxes of all interested atoms at time *t*, *N* is the number of the atoms, $q_{j}$ is the fixed electric charge of the jth atom and $\boldsymbol{v}_{j}(t)$ is the velocity vector of the jth atom at time *t*. With the MD simulation data of the DNA fragment, we can calculate its absorption spectrum according to equation (3). In addition, to better determine the peaks of the N-H vibration, we exclusively calculated the absorption spectrum of the lysine and arginine amino groups. Based on Suppl. Figure 2a-b and the experimental spectrum we identified three central vibrational frequencies for N-H bonds. To be specific, the weakly H-bonded N-H vibrations are located at 3433 cm^-1^ (asymmetric stretching) and 3300 cm^-1^ (symmetric stretching), respectively, while the strongly H-bonded N-H vibrations are located at 2746 cm^-1^. The corresponding experimental wavenumbers are 3330, 3264, and 2769 cm^-1^, respectively with an acceptable shift. Theoretically, an THM radiation at each of the aforementioned three frequencies could resonate with the N-H bond and alter the related H-bond connections between the protein and DNA. Nevertheless, the first two reside in the region of strong water absorption and thus hardly penetrate water surroundings and reach the nucleosome. Hence, the one at 2,746 cm^-1^ corresponding to the strongly H-bonded N-H vibrations could be most effective to alter the histone-DNA interactions.

1. **THM mostly promoted electrostatic attractions between DNA and lysine and arginine residues**

In simulations, when the nucleosome was irritated by a 2746 cm^-1^ (3.64 μm) [Terahertz](javascript:;) [wave](javascript:;) light, the binding free energy between the histones and DNA decreased by 4,206 kJ/mol, which suggested an enhanced affinity within the complex. Figure 2e indicated that the binding enhancement mainly came from the alteration of the electrostatic contribution, as the electrostatic free energy decreased by 4,999 kJ/mol in response to the irritation. Therefore, the [Terahertz](javascript:;) [wave](javascript:;) radiation must reinforce the electrostatic bonding between the DNA and histones. The variations of affinity brought by the van der Waals interactions and non-electrostatic solvation were insignificant. But it was not the case for the polar solvation, which is the second evidence that the electrostatic interactions changed most under radiation.

As given in Suppl. Figure 2c, variation of the binding free energy was decomposed based on all residues involved. Apparently, the free energies altered by the lysine and arginine were significantly higher than by other amino acids. They in total decreased the binding free energy by over 3000 kJ/mol when irritated. The DNA components (dA/T/G/C) ranked the second with each decreasing the free energy by over 500 kJ/mol. Other amino acids were less contributive to the enhanced binding, or even weakened the binding such as the negatively charged glutamic and aspartic acids. This was understandable as they competed with DNA in forming H-bonds with lysine and arginine. We can now conclude that the Terahertz wave radiation must have promoted the electrostatic attractions between the DNA and lysine and arginine, which plays a dominant role in DNA-histones binding.

1. **THM enhanced binding via the specific N-H bond**

To further determine if the [Terahertz](javascript:;) [wave](javascript:;) radiation enhanced the binding via the specific N-H bond, we introduced radiations with different frequencies into the complex and summarized variations of the binding energies. Here we only consider the frequency region of 2400-4000 cm^-1^ based on Fourier Transform Infrared (FTIR) spectroscopy studies [2]. Suppl. Figure 2d demonstrated that, the DNA-histones binding is enhanced locally most under THM radiations at central frequencies of the strongly H-bonded N-H vibrations (2746 cm^-1^) and the weakly H-bonded symmetric N-H stretching (3300 cm^-1^). The further the irritation frequency deviated locally from either of the two central frequencies, the less the radiation contributes to the affinity. Although the radiation at 3300 cm^-1^ gave rise to the largest binding enhancement, it is not applicable in the real world due to strong absorption by water surroundings. As a consequence, in experiments in order to effectively change the gene expression, the radiation frequency is expected to switch in the vicinity of 2746 cm^-1^ (3.64 μm).

1. **THM barely altered conformations of the DNA or protein**

In the main text we have only considered the interactions between the protein and the DNA with or without [Terahertz](javascript:;) [wave](javascript:;) illumination. There might be some conformational changes of the individuals once irritated, which could also alter the binding. Here we firstly calculated the numbers of H-bonds connecting two single DNA chains with and without the field irritation at 2746 cm^-1^ (3.64 μm) (Suppl. Figure 2e). As expected, the number of strong hydrogen bonds changed trivially from 360.4 ± 5.5 to 361.7 ± 5.2. That is, there was almost no H-bond destruction and reconstruction between the DNA double chains. Thus, the primary structure of the DNA itself was barely altered by the 3.64 μm [wave](javascript:;) radiation and there was no DNA unwinding. It suggested that this stimulation would not cause base-pair mismatch and gene mutation. This is consistent with our previous results [5]. Furthermore, the DNA conformation changed insignificantly under this specific [terahertz](javascript:;) [wave](javascript:;) irritation, which denoted a stable second/tertiary structure.

In addition, we have calculated the number of H-bonds in the protein and its root mean square deviation (RMSD, Suppl. Figure 2e-f). There was an increase of the H-bond number under irritation from 662.4 ± 13.6 to 666.4 ±11.1, which nevertheless indicated a more stable protein structure with stronger non-bonded binding among amino acides. This agrees well with the trivial conformational change of irritated protein. To conclude, the [Terahertz](javascript:;) [wave](javascript:;) radiation although effective on the DNA-protein binding did not change the individual conformations significantly. Hence, [Terahertz](javascript:;) [wave](javascript:;) stimulation is predicted to be a safe therapeutic approach.

**Supplementary Figures**

**Figure S1. Hydrogen bonds within the DNA and salt bridges between histones and the DNA.**

**
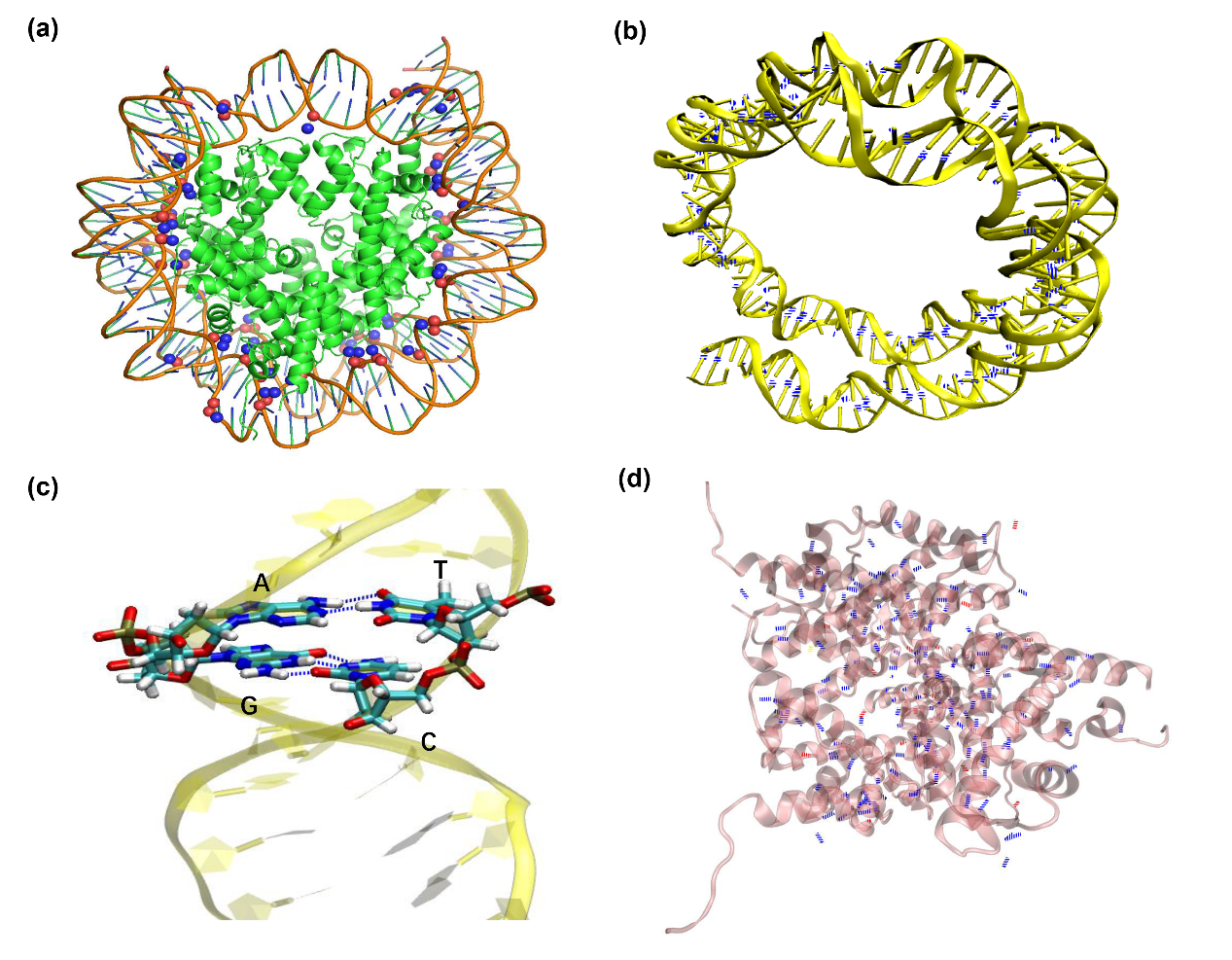
**

**Figure S1.** (a) O atoms (red spheres) in PO_4_^-^ of the DNA and N atoms (blues spheres) in LYS and ARG positively charged side chains where the N-O pair distances are within 4 Å, which is the maximum distance allowed for a salt bridge to be formed. (b) Peripheral DNA structure (yellow) of the nuclesome core particle where the hydrogen bonds connecting the paired pyrimidine-purine nucleotide bases are indicated in dashed blue lines. (c) N—H···O and N—H···N bonds (dashed blue lines) connecting the thymine (T) and cytosine (C) with, respectively, the adenine (A) and guanine (G). (d) Histones of the nuclesome core particle. The blue, red and yellow dashed lines indicate the N—H···O, O—H···O, and S—H···O bonds, respectively connecting the amino acides.

**Figure S2. Mechanism underlying the enhanced histones-DNA binding.**

**
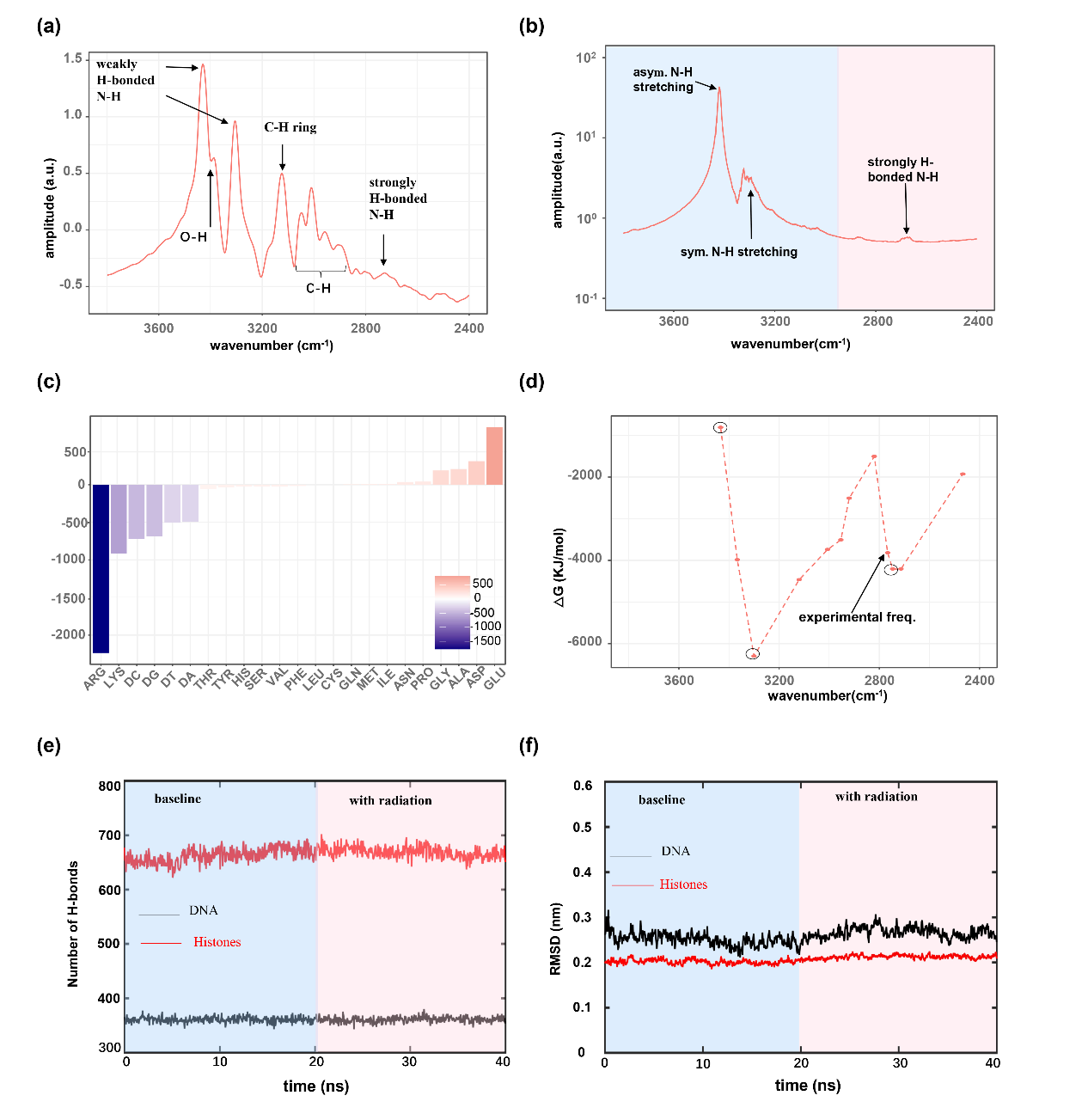
**

**Fig S2.** (a) Absorption spectrum of the DNA unit in the region 2400-3800 cm^-1^. (b) Absorption spectrum of N-H bonds. The vibrational modes of observable absorption peaks are indicated (the region of strong water absorption is masked in blue, and the region of weak water absorption is masked in red). (c) Residue contributions to the binding free energy variation after being irritated. (d) Bind free energy variations between the baseline and the irritated cases vs. Terahertz wave radiation wavenumber. The three circled frequencies correspond to the vibrational modes indicated in Fig 2e). (e) Numbers of hydrogen bonds in the DNA and histones before and after the THM. (f) RMSD of the DNA and histones before and after the terahertz wave irritation.

**Figure S3.** Effects of THM (0.3 mW) on HCT-8 cell

**
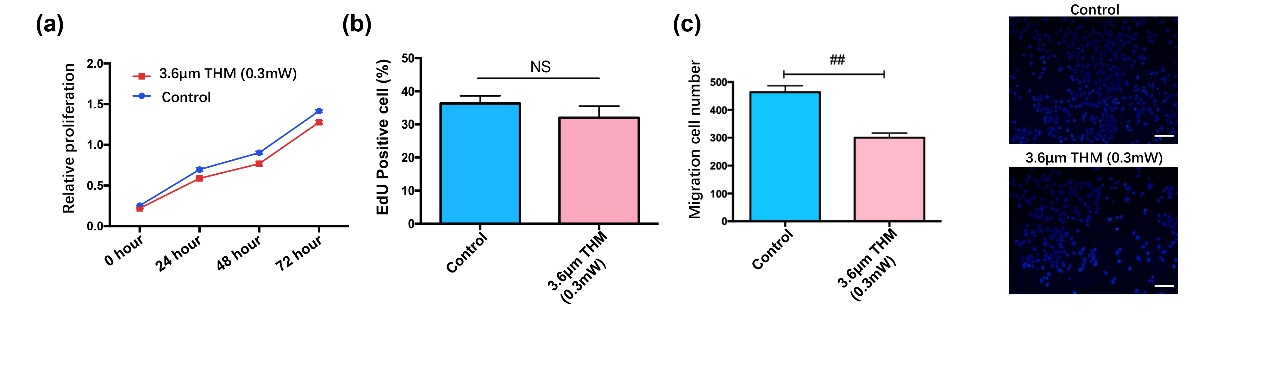
Fig S3.** (a-b) Effects of THM (3.6 μm) on HCT-8 cell proliferation and growth. (c) The effect of THM (λ= 3.6 μm) on cell migration. Scale bar, 50 μm (right). (All data shown as mean ± SEM, ##p<0.01 NS: not significant).

| Gene name | Primer Sequences |
| --- | --- |
| GAPDH | F: AATGGGCAGCCGTTAGGAAA  R: GCCCAATACGACCAAATCAGAG |
| CPQ | F: GATGGGGGCAAAGACCTACC |
|  | R: TTTGGACGCAGCCCAAGAT |
| ABHD5 | F: AATTCTGGCACCAGCATCCA |
|  | R: GTCACCAGAAAGCACACAGGT |
| ABI3BP | F: CTTGAACAGCCAAGGGCAAC |
|  | R: GGCACTTGTGGTTCTTTCAGG |
| RBMS3 | F: GTCTCCACATACCAGGGTGC |
|  | R: ACACAACAGCTGGTGGAGTA |
| CGNL1 | F: TGAGCTTGCGTTTGAAAGCC |
|  | R: TCGGCAGCTTCTTCAGTCTTA |
| ITGA5 | F: GCTTCAACTTAGACGCGGAG |
|  | R: GCACACTGACCCCGTCTG |
| TMOD2 | F: ACAACATTAAGGCTTTTGCAGACA  R: TGCTGCCTCTGGTTGTCAAT |
| RTN4R | F: GAATGTGCTGGCCCGAATTG |
|  | R: CAGACCGGAGCTGTGCATTA |
| CCBE1 | F: TAGCACATCCCAACACCGTC  R: GCCTAGGAGGGGACTCTGAA |

**Table S1. RT-qPCR primers used in this study.**

**References**

1. Movasaghi, Z., S. Rehman, and D.I. ur Rehman, *Fourier Transform Infrared (FTIR) Spectroscopy of Biological Tissues.* Applied Spectroscopy Reviews, 2008. **43**(2): p. 134-179.

2. Dovbeshko, G.I., et al., *FTIR spectroscopy studies of nucleic acid damage.* Talanta, 2000. **53**(1): p. 233-46.

3. Agarwal, V., et al., *Simulating infrared spectra and hydrogen bonding in cellulose Ibeta at elevated temperatures.* J Chem Phys, 2011. **135**(13): p. 134506.

4. Praprotnik, M. and D. Janezic, *Molecular dynamics integration and molecular vibrational theory. II. Simulation of nonlinear molecules.* J Chem Phys, 2005. **122**(17): p. 174102.

5. Wu, K., et al., *Terahertz Wave Accelerates DNA Unwinding: A Molecular Dynamics Simulation Study.* J Phys Chem Lett, 2020. **11**(17): p. 7002-7008.
